# Immunohistochemiocal subtyping using CK20 and CK5 can identify urothelial carcinomas of the upper urinary tract with a poor prognosis

**DOI:** 10.1371/journal.pone.0179602

**Published:** 2017-06-20

**Authors:** Danijel Sikic, Bastian Keck, Sven Wach, Helge Taubert, Bernd Wullich, Peter J. Goebell, Andreas Kahlmeyer, Peter Olbert, Philipp Isfort, Wilhelm Nimphius, Arndt Hartmann, Johannes Giedl, on behalf of the Bridge Consortium

**Affiliations:** 1 Department of Urology and Pediatric Urology, University Hospital Erlangen, Erlangen, Germany; 2 Department of Urology and Pediatric Urology, Philipps University of Marburg, Marburg, Germany; 3 Institute of Pathology, Philipps University of Marburg, Marburg, Germany; 4 Institute of Pathology, University Hospital Erlangen, Erlangen, Germany; Centro Nacional de Investigaciones Oncologicas, SPAIN

## Abstract

**Purpose:**

Genome-wide analyses revealed basal and luminal subtypes of urothelial carcinomas of the bladder. It is unknown if this subtyping can also be applied to upper tract urothelial carcinomas.

**Materials and methods:**

Tumor samples from 222 patients with upper tract urothelial carcinomas who were treated with radical nephroureterectomy were analyzed for the expression of seven basal/luminal immunohistochemical markers (CK5, EGFR, CD44, CK20, p63, GATA3, FOXA1).

**Results:**

Hierarchical clustering revealed a basal-like subtype (enrichment of CK5, EGFR and CD44) in 23.9% and a luminal-like subtype (enrichment of CK20, GATA3, p63 and FOXA1) in 13.1% of the patients. In 60.8%, little to no markers were expressed, whereas markers of both subtypes were expressed in 2.2%. By using CK5 and CK20 as surrogate markers for the basal and luminal subtypes, we defined four subtypes of upper tract urothelial carcinomas: (i) exclusively CK20 positive and CK5 negative (CK20+/CK5-), (ii) exclusively CK5 positive and CK20 negative (CK20-/ CK5+), (iii) both markers positive (CK20+/CK5+) and (iv) both markers negative (CK20-/CK5-). A receiver-operator analysis provided the optimal cut-off values for this discrimination. An immunoreactive score >1 for CK5 and >6 for CK20 were defined as positive. In multivariate Cox’s regression analysis, the CK20+/CK5- subtype was an independent negative prognostic marker with a 3.83-fold increased risk of cancer-specific death (p = 0.02) compared to the other three subtypes.

**Conclusions:**

Immunohistochemical subgrouping of upper tract urothelial carcinomas by analyzing CK5 and CK20 expression can be performed in a routine setting and can identify tumors with a significantly worse cancer-specific survival prognosis.

## Introduction

Upper tract urothelial carcinoma (UTUC) is a rare tumor entity accounting for only 5% of all urothelial carcinomas [1]. Although organ-confined UTUC can be efficiently treated by radical nephroureterectomy and resection of the ipsilateral bladder cuff, advanced and metastatic UTUC remains a significant clinical problem with a median survival of only six months for patients with T4 tumors [2, 3]. Despite limited data on systemic therapy for advanced UTUC, guideline recommendations are similar to those for advanced urothelial carcinoma of the bladder (UCB) [4]. Because treatment options for metastatic UCB have made limited progress in the last two decades, platin-based chemotherapies remain the most relevant first-line therapy [5–7]. However, recent studies demonstrated that genes and genomic regions are either mutated or altered frequently in UCB, which could be relevant for future targeted therapies [8–11]. The PD-1/PD-L1 complex is currently the most important example of molecular-based immunotherapeutic treatment, which led to the approval of the checkpoint inhibitor atezolizumab by the FDA as a 2^nd^-line treatment against metastatic UCB that progressed after platin-based chemotherapy [12]. Moreover, several studies showed that based upon genetic expression patterns, UCB can be classified into different molecular subtypes with distinct clinical features regarding survival and response to chemotherapy. This opens the possibility to individualize treatment options according to the molecular profile of the tumor [13–17]. Molecular subtyping of UCB is reminiscent of molecular subtyping of breast cancer, in which the analysis of gene expression patterns over the last 15 years resulted in the discovery of four different subtypes (luminal A, luminal B, *ERBB2*-overexpression, and basal-like) that have different responses to chemotherapy, endocrine therapy or anti-HER2 therapy. These established molecular subtypes can be diagnosed with convenient approximation using immunohistochemistry for the expression of only four markers (estrogen receptor, progesterone receptor, human epidermal growth factor receptor 2 (HER2), and Ki-67) [18, 19]. These findings revolutionized the treatment of breast cancer, and a similar impact can be expected for UCB, as highly similar molecular subtypes were identified. However, to date, it is unknown whether these molecular subtypes and their prognostic impact exist in UTUC and whether such subtypes could be easily classified using immunohistochemistry. Therefore, we assessed the immunohistochemical expression of characteristic proteins with the potential to stratify UTUC into basal-like and luminal-like subtypes and to evaluate the prognostic impact of these alterations in a retrospective series of UTUC.

## Materials and methods

### Patients

A total of 222 patients diagnosed with UTUC at a median age of 72 years (interquartile range (IQR) 66–78 years) and treated with radical nephroureterectomy at the Departments of Urology at University Hospital Erlangen and Philipps University of Marburg between 1996 and 2014 were retrospectively analyzed. The analysis was conducted in November 2016. The study was performed according to the standards established in the Declaration of Helsinki under a positive vote of the institutional review board of the University of Erlangen-Nuremberg. Written informed consent was obtained from the patients.

### Immunohistochemical analysis

All tumor cases were typed, graded and staged by two experienced uropathologists (AH, JG) according to the latest edition of the TNM/AJCC classification and the 2016 WHO classification. In addition, grading according to the 1973 WHO classification was performed. A tissue microarray (TMA) comprising the 222 tumor cases was constructed (core diameter 1.5 mm, one tumor core/patient). Seven different antibodies were used against markers that were predicted to discriminate between basal and luminal-like expression patterns. For the basal-like expression pattern, primary antibodies against the cytokeratin CK5 (Zytomed, clone XM26, 1:50 dilution), the cell-surface adhesion molecule CD44 (Dako, clone DF1485, 1:40 dilution), the transcription factor p63 (DCS, clone SFI-6, 1:100 dilution) and the epidermal growth factor receptor EGFR (Novocastra, clone EGFR.25, 1:50 dilution) were used. For the luminal-like expression pattern, primary antibodies against CK20 (Dako, clone Ks20.8, 1:50 dilution) and the transcription factors FOXA1 (Abcam, polyclonal, 1:500 dilution) and GATA3 (DCS, clone L50-823, 1:1000 dilution) were used.

All immunohistochemical stains were scored using a semiquantitative immunoreactive score (IRS), quantifying staining intensity (0 (no staining reaction), 1+ (weak staining reaction), 2+ (moderate staining reaction), 3+ (strong staining reaction)) and percentage of positively stained cells (0 (0%), 1 (<10%), 2 (10–50%), 3 (51–80%), 4 (>80%)) resulting in IRS values ranging from 0 to 12 [20].

### Reduction of the marker panel

By analyzing the expression patterns of the aforementioned seven markers, we wanted to investigate the existence of basal and luminal subtypes in UTUC. As a set of seven immunohistochemical markers is not easily applicable in a daily routine setting, we tried to recreate the results gained with seven markers with just two surrogate markers. Therefore, CK5 and CK20 were chosen as prototypic markers for basal-like and luminal-like subtypes, respectively, since both markers are widely used in routine surgical pathology diagnosis and demonstrate an inverse expression pattern [15, 21]. Moreover, CK5 and CK20 have previously been already used for subtyping of UCB [22–24].

### Statistical analyses

Hierarchical clustering of the IRS values was performed using Euclidean distance and complete-linkage clustering. Comparisons of the continuous variables were conducted using non-parametric Kruskal-Wallis statistical tests, and comparisons of the categorical variables were conducted using Chi-squared statistical tests. The differences in the patients’ survival times were examined using the Kaplan-Meier method and log-rank statistics. The relative risks for patient survival were established by fitting multivariate Cox’s regression models. Optimized immunohistochemical cut-off values for discrimination between patients that suffered a cancer-related death and patients that did not were defined by receiver operating characteristic (ROC) analyses using cancer-specific survival as the response variable and IRS values as predictors. All calculations were performed with the R statistical framework Ver. 3.2.1 (R Foundation for Statistical Computing, Vienna, Austria. http://www.R-project.org/).

## Results

Table 1 shows the clinicopathological characteristics of the patients. Our study cohort comprised 149 male and 73 female patients who received a nephroureterectomy. Lymphadenectomy was performed only when lymph node metastases were clinically suspected. Patients were followed up for a median period of 16 months (IQR 4–61 months). A total of 101 patients died during the observation period, with 52 of those deaths because of cancer-related causes.

**Table 1:** Patient characteristics.

Gender, n (%)		
	Male	149 (67.1)
	Female	73(32.9)
Age at diagnosis, years (IQR)		
	Median	72 (66–78)
Follow up, months (IQR)		
	Median	16 (4–61)
Survival, n (%)		
	Disease specific death	52 (23.4)
	Other causes of death	49 (22.1)
Tumor stage, n (%)		
	pT1	35 (15.8)
	pT2	27 (12.2)
	pT3	91 (41.0)
	pT4	29 (13.1)
	pTa	38 (17.1)
	pTis	0 (0)
	NA	2 (0.9)
Tumor grade (WHO 1973), n (%)		
	G1	0 (0)
	G2	95 (42.8)
	G3	127 (67.2)
Tumor grade (WHO 2004), n (%)		
	Low grade	57 (25.7)
	High grade	165 (74.3)
Lymph node status, n (%)		
	N0/Nx	156 (70.3)
	N1	45 (20.3)
	NA	21 (9.5)
Metastases, n (%)		
	M0/Mx	163 (73.4)
	M1	26 (11.7)
	MA	33 (14.9)

**Fig 1:**
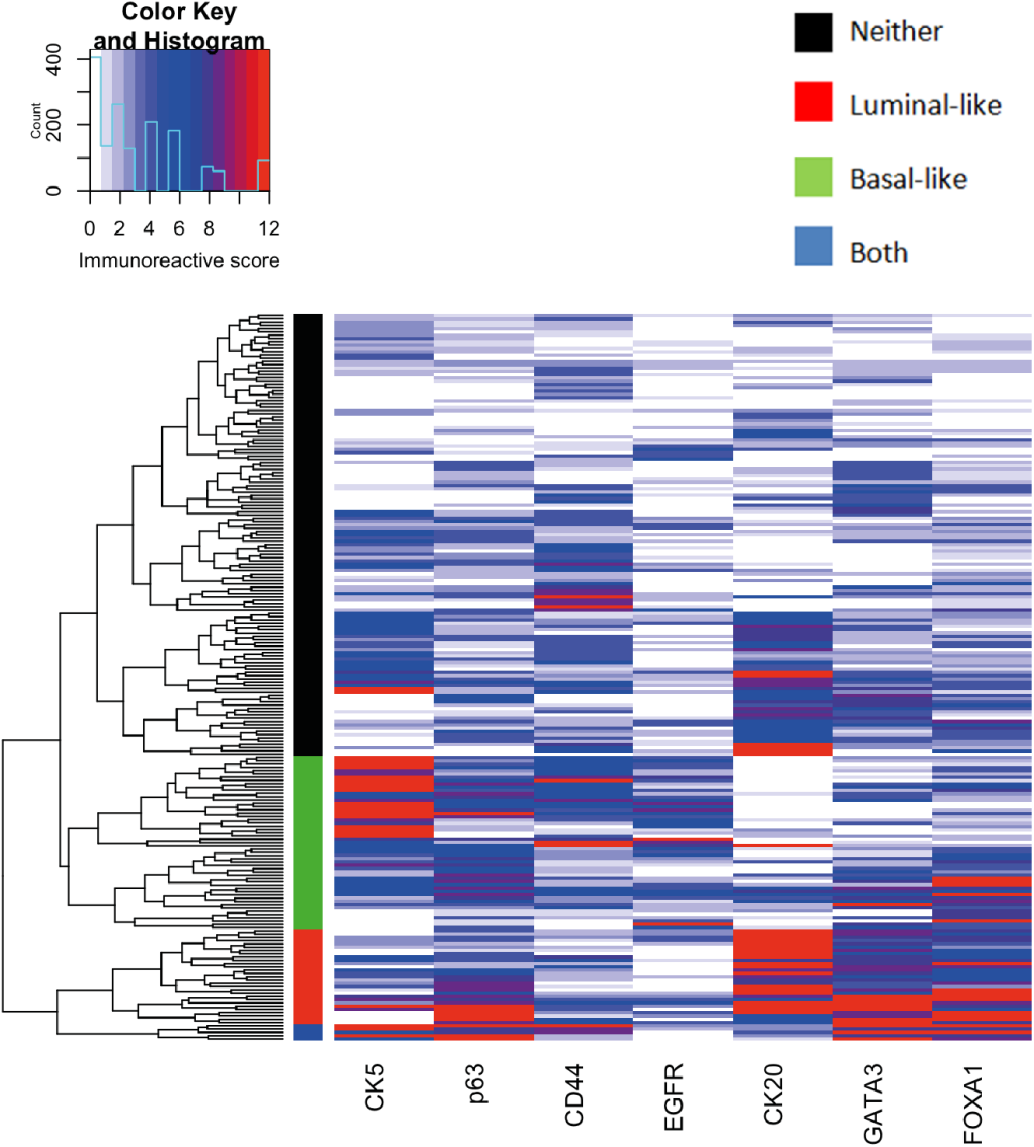
Heatmap and cluster dendrogram demonstrating the expression patterns of the seven analyzed markers in UTUC. Using hierarchical clustering the tumor samples could be classified into four groups based on expression of the markers: A basal-like subtype with high expression levels of CK5, CD44 and, to a lesser extent, EGFR (green cluster); a luminal-like subtype with high expression levels of CK20, GATA3 and FOXA1 (red cluster); a subtype with expression of both luminal and basal-type markers (blue cluster); a subtype without significant expression of any markers (black cluster).

At first, we aimed at characterizing our patient cohort with respect to the expression of the selected seven basal-like or luminal-like markers (S1 Table). After hierarchical clustering, we visually identified four distinct clusters of samples. The row dendrogram, representing the distance between individual tumor samples was cut so that the visually identified clusters could be distinguished (Fig 1). In 53 patients (23.9%), there was a predominantly basal-like expression pattern of the selected markers, which were characterized by high expression levels of CK5, CD44 and, to a lesser extent, EGFR. In 29 patients (13.1%), a predominantly luminal-type pattern of markers, including high expression levels of CK20, GATA3 and FOXA1, was found. Clustering showed p63 enrichment primarily in the luminal-like subtype. A group of 135 patients (60.8%) did not display any particular expression pattern that could be linked to either basal or luminal-like type and was classified as neither basal nor luminal. Interestingly, a fourth group, comprising 5 cases (2.2%) showed expression of both luminal and basal-type markers.

This approach, however, was not successful to stratify the patient cohort in terms of cancer-specific survival (p = 0.23; log-rank test, S1 Fig).

**Fig 2:**
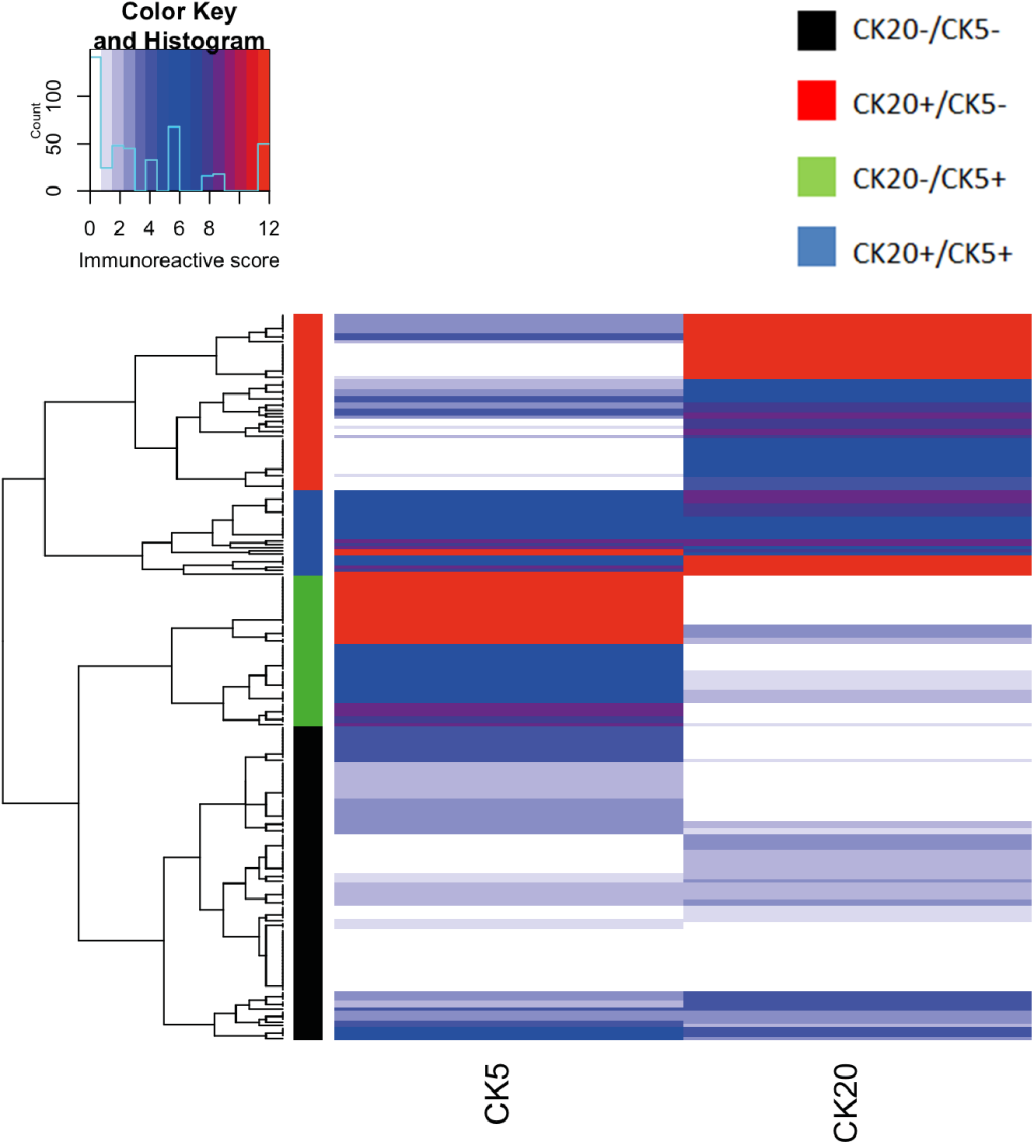
Heatmap and cluster dendrogram demonstrating the expression patterns of CK5 and CK20 in UTUC. Using CK5 and CK20 as surrogate markers for the subtypes, hierarchical clustering again found four subtypes: a basal-like subtype with high CK5 expression (green cluster), a luminal-like subtype with high CK20 expression (red cluster); a subtype with expression of CK5 and CK20 (blue cluster); a cluster without a predominant expression of CK5 nor CK20 (black cluster).

As a set of seven immunohistochemical markers is not easily applicable in a daily routine setting, we tried to recreate the results gained with seven markers with just two surrogate markers (CK5 for basal-like and CK20 for luminal-like subtype). We applied the same strategy as before, consisting of hierarchical clustering, visual identification of sample clusters and classification based on the sample dendrogram. This way, the identification of four subgroups was still possible (Fig 2). The patient group with high basal-like CK5 expression was reduced to 43 cases (20.7%), and the number of luminal-like cases classified with high CK20 expression increased to 54 cases (24.4%). The patient group that could previously not be clearly classified decreased in number, with 96 patients (43.2%) presenting without any predominant basal or luminal-like markers and 26 cases (11.7%) showing high expression of both markers.

We also tested for an association of the defined clusters of patients with cancer-specific survival. As before, we did not detect a significant difference in cancer-specific survival between the defined patient groups (p = 0.54, log-rank test, S2 Fig). In summary, using the protein expression of a defined set of selected basal or luminal-like markers, it is possible to define tumor samples that can be regarded as basal-like or luminal-like. However, these sample clusters, defined by hierarchical clustering and visual classification were not associated with patient’s survival. Also, an exact classification was hampered by a fluent passage between the four subtypes.

However, the expression of CK5 and CK20 has already been successfully used as a predictor of patient survival in UCB [24] Hereby, an approach of defining distinct cut-off values for both markers has proven valuable for defining patients with a low or high risk of a cancer-specific death. In order to test for a specific association of staining patterns of CK5 and CK20 with prognosis, we additionally applied a data-driven approach and sought to define patient subgroups with a low or high risk of a cancer-specific death. Therefore, distinct cut-off values of the IRS of CK5 and CK20 were defined that allowed an optimal discrimination regarding cancer-related survival. A ROC analysis provided the ideal cut-off values. A positive status was defined as an IRS >1 for CK5 and an IRS >6 for CK20. We validated the results of these selection procedures by bootstrapping. The results of the internal validation are presented as supplementary data (S2 Table).

Using these cut-off values, the four subgroups were defined as follows: Exclusive positivity for CK5 (CK20-/ CK5+) in 117 patients (52.7%), exclusive positivity for CK20 (CK20+/CK5-) in 16 patients (7.2%), positive for both markers (CK20+/CK5+) in 32 patients (14.4%), and negative for both markers (CK20-/CK5-) in 57 patients (25.7%). The composition of the subtypes showed differences depending on whether they were defined by heatmap or IRS cut-off values. The subtypes defined by the IRS cut-off values were significantly associated with tumor stage (p<0.01) and grade according to the WHO classifications of 1973 and 2004 (p = 0.03; p<0.01). No association was found with gender, lymph node status and presence of metastases (Table 2).

**Table 2:** Distribution of subtypes of UTUC.

		CK20-/CK5+	CK20+/CK5-	CK20-/CK5-	CK20+/CK5+	p
Gender, n (%)						
	Male	82 (70.1)	12 (75.0)	35 (61.4)	20 (62.5)	0.56
	Female	35 (29.9)	4 (25.0)	22 (38.6)	12 (37.5)	
Age (years)						
	Median (IQR)	73 (66–78)	70.5 (68–76)	70 (65–76)	72 (67–79)	0.58
Tumor stage, n (%)						
	pTa/pT1	36 (31.0)	1 (6.2)	16 (28.6)	20 (62.5)	**<0.01**
	pT2	9 (7.8)	3 (18.8)	10 (17.8)	5 (15.6)	
	pT3	54 (46.5)	10 (62.5)	22 (39.3)	5 (15.6)	
	pT4	17 (14.7)	2 (12.5)	8 (14.3)	2 (6.3)	
Grade (WHO 1973), n (%)						
	G2	52 (44.4)	5 (31.3)	18 (31.6)	20 (62.5)	**0.03**
	G3	65 (55.6)	11 (68.7)	39 (68.4)	12 (37.5)	
Grade (WHO 2004), n (%)						
	Low grade	32 (27.4)	1 (6.3)	7 (12.3)	17 (53.1)	**<0.01**
	High grade	85 (72.6)	15 (93.7)	50 (87.7)	15 (46.9)	
Lymph nodes, n (%)						
	pN0/pNx	83 (79.0)	10 (66.7)	37 (71.2)	26 (89.7)	0.18
	pN+	22 (21.0)	5 (33.3)	15 (28.8)	3 (10.3)	
Metastases, n (%)						
	pM0/pMx	84 (85.7)	10 (76.9)	41 (83.7)	28 (96.6)	0.28
	pM+	14 (14.3)	3 (23.1)	8 (16.3)	1 (3.4)	

**Fig 3:**
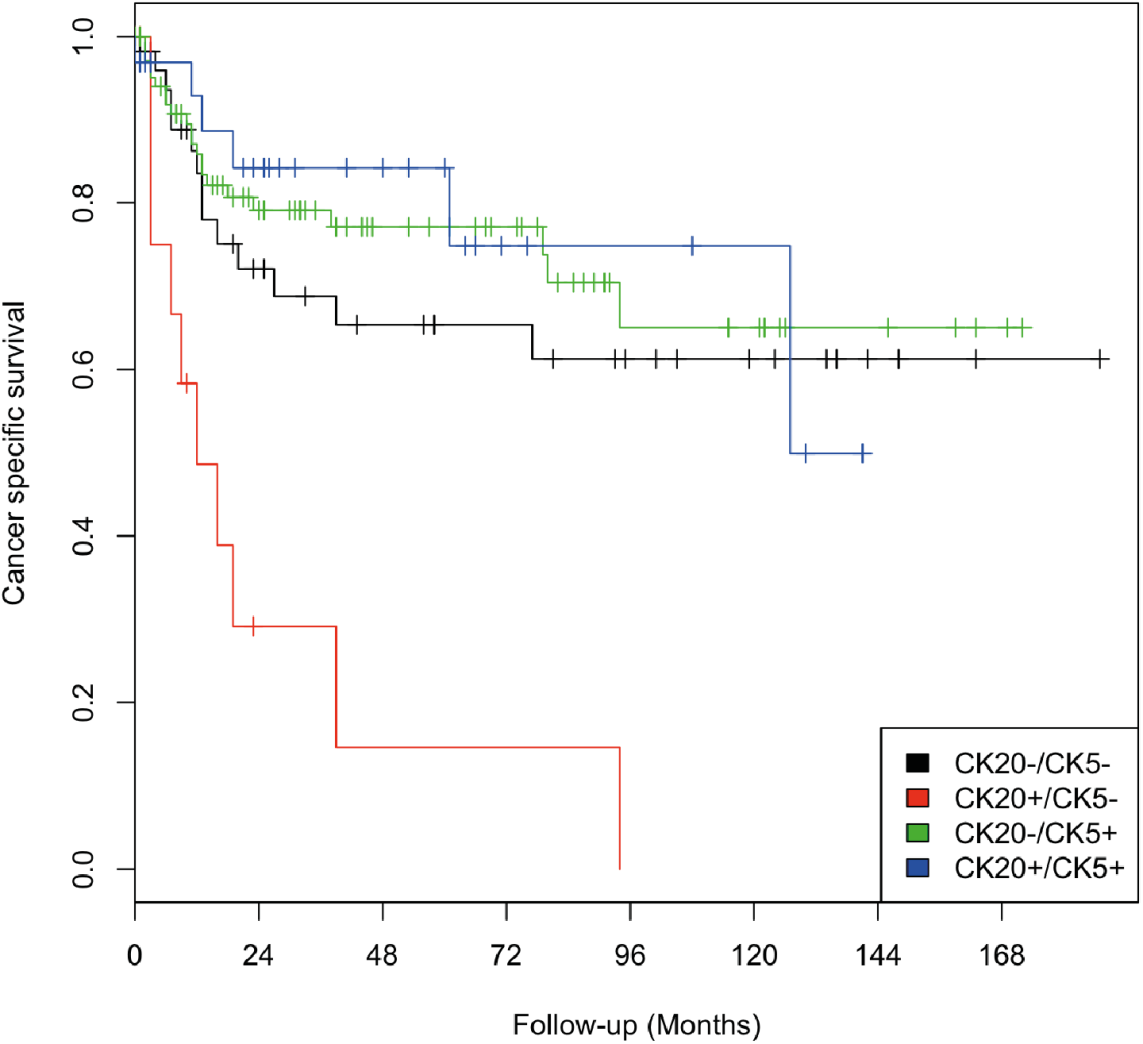
Cancer-specific survival depending on subtype of UTUC defined by CK5 and CK20 expression using IRS cut-off values determined by ROC analysis. Using ROC analysis the optimal IRS cut-off values for CK5 and CK20 were defined. IRS scores >1 for CK5 and >6 for CK20 were defined as high expression. Using these cut-off values to define the four subgroups, the CK20+/CK5- subtype showed a significantly worse cancer-specific survival when compared to the other subtypes.

Kaplan-Meier analysis confirmed the significantly worse cancer-specific survival rates in the group exclusively positive for CK20 (CK20+/CK5-). The three remaining groups (CK20-/CK5+, CK20+/CK5+, and CK20-/CK5-) showed no significant difference in survival (Fig 3).

In the multivariate Cox’s regression analysis, this specific immunohistochemical expression pattern of exclusive positivity for CK20 (CK20+/CK5-) was an independent prognostic predictor with a 3.83-fold increased risk of tumor-specific death (p = 0.02) (Table 3).

**Table 3:** Multivariate Cox’s regression analyses predicting cancer-specific mortality.

Covariate		Relative risk (95% confidence interval)	p
Marker expression			
	CK20-/CK5-	Reference	
	CK20-/CK5+	0.71 (0.30–1.68)	0.43
	CK20+/CK5-	3.83 (1.29–11.37)	0.02
	CK20+/CK5+	0.97 (0.32–2.87)	0.95
Gender			
	Female	Reference	
	Male	0.75 (0.37–1.54)	0.44
Tumor stage			
	pTa/pT1	Reference	
	pT2	4.52 (1.04–19.65)	0.04
	pT3	4.04 (1.04–15.71)	0.04
	pT4	8.21 (1.87–35.96)	<0.01
Grade (WHO 2004)			
	Low grade	Reference	
	High grade	1.43 (0.44–4.61)	0.55
Lymph nodes			
	pN0/pNx	Reference	
	pN+	1.32 (0.54–3.24)	0.54
Metastases			
	pM0/pMx	Reference	
	pM+	5.17 (2.39–11.15)	<0.01

## Discussion

Recent molecular investigations have dramatically changed the perception of urothelial carcinomas. It has been shown that UCB has a very high mutation rate exceeded only by those of lung carcinoma and melanoma [14]. The Cancer Genome Atlas Research Network has identified 32 recurring genetic mutations in UCB, including mutations of genes involved in cell cycle regulation, chromatin regulation and kinase signaling pathways [8]. These mutations are of prognostic and therapeutic value, as mutations of some genes, such as *FGFR3* and *TP53* appear to be tumor-stage dependent, whereas others, including mutations in the phosphatidylinositol-3-OH kinase/AKT/mTOR pathway, could be targets for possible future therapies [8]. Equally important is the discovery of molecular subtypes in UCB, which has been demonstrated by several independent studies using gene expression pattern analysis. Although there are still many uncertainties regarding the number and clinical features of these subtypes, most studies agree on the existence of a basal and luminal subtype analogous to breast cancer [14–16]. Basal and luminal UCBs are characterized by distinct genetic patterns. Luminal UCB usually shows increased expression of CK19 and CK20 and certain transcription factors associated with hormonal receptors (GATA3, FOXA1) as well as mutations in the fibroblast growth factor receptor 3 gene (*FGFR3*). By contrast, basal tumors are characterized by an enrichment for the cytokeratins CK5 and CK14, certain transcription factors (SNAI, TWIST) and increased expression of the cell surface glycoprotein CD44 as well as increased expression of EGFR [15, 16]. Subtyping of UCB is also clinically relevant, because basal-like bladder cancers show significantly decreased overall and cancer-specific survival, while luminal bladder cancers have been suggested to have a worse response to chemotherapy, although this is still under debate [25, 26].

However, it is still unclear to what extent these findings can be translated to UTUC. The rare incidence of UTUC makes investigations of large cohorts difficult, leading to the application of the therapeutic strategies for UCB on advanced UTUC as well. Although UTUC has been found to have similar genetic mutations as UCB [27], a recent genomic characterization of UTUC by Sfakanios et al. [28] reported significant differences in the prevalence of certain mutations, e.g., *FGFR3* mutations are more common in UTUC whereas mutations in *TP53* are more common in UCB. Moreover, UTUC seems to have distinct pathogenetic features as, unlike UCB, it is associated with a hereditary predisposition within the hereditary nonpolyposis colorectal cancer syndrome [29] and frequently shows microsatellite instability and loss of mismatch repair proteins [30].

In this study, we investigated UTUC to identify (molecular) subtypes using immunohistochemistry. To our knowledge molecular subtyping of UTUC has not been performed to this extent before. Using seven immunohistochemical markers associated with basal and luminal subtypes of UCB, the existence of four distinct subgroups in UTUC could be delineated. Surprisingly, apart from the basal-like subtype, which showed increased expression of CK5, EGFR and CD44 and the luminal-like subtype, which showed increased expression of CK20, GATA3 and FOXA1, few tissue samples showed enrichment of basal and luminal-like markers, whereas 60.8% of the tested samples showed no marker expression of either basal or luminal subtypes. This is in contrast to UCB, in which most tumors can be assigned to either the basal-like or luminal-like subtype [16]. Interestingly, hierarchical clustering showed an association of p63 with luminal markers in UTUC although p63 usually controls MYC expression in human bladder cancer cells, which is enriched in basal tumors [31].

Since the evaluation of seven immunohistochemical markers is impractical in daily clinical routines, reducing the marker panel to only the two most characteristic markers CK5 and CK20 as surrogate markers for basal and luminal-like urothelial carcinomas, respectively, was performed. This allowed for the identification of the four previously described subtypes; however, because there were some differences compared to the analysis using all seven markers, the subtypes were referred to as exclusively CK5 positive (CK20-/CK5+), exclusively CK20 positive (CK20+/CK5-), both CK20 and CK5 positive (CK20+/CK5+) and marker negative (CK20-/CK5-) subtypes. The most striking finding in this study is the significantly worse cancer-specific survival of the exclusively CK20 positive group (CK20+/CK5-) compared to the other three subgroups. Even more interesting is the finding that the subtype with high positivity for both CK5 and CK20 (CK20+/CK5+) was not associated with reduced survival. This might suggest a “protective” role of either CK5 or other basal markers in CK20 positive patients. These findings are in contrast to previous results in muscle-invasive bladder cancer (MIBC) and breast cancer, in which basal tumors are associated with significantly worse outcomes [16]. Our results are also in contrast to a recent international multicenter study by Raman et al., who reported an association between worse survival and decreased FOXA1 expression, which is akin to basal urothelial carcinomas [32]. In our cohort, no association between survival and subtyping of UTUC was found when using FOXA1 as the only marker. A possible reason for the different results might be that Raman et al. used the Allred scoring system to quantify marker expression, whereas this study used the IRS. Another reason for the discrepancies might be that in both studies only one or very few specific markers were used as surrogate markers for the UTUC subtypes. Additional tests with other markers might offer different results. Our results are however in agreement with a recently published comprehensive transcriptional analysis of 460 early-stage UCB and 16 MIBC samples revealing 3 distinct classes of UCB [21]. The so-called class 2, which is characterized by a poor prognosis and was found in 14 of the 16 investigated MIBCs, showed high CK20 expression. In our study almost all of the exclusively CK20 positive (CK20+/CK5-) UTUC cases were at least stage pT2.

There are several limitations to our study. First, there is the limitation of a retrospective study. Because of missing data, the exact location of the UTUC was unknown in some cases. Moreover, no differentiation was made regarding variant morphologies of UTUC. Additionally, although seven markers were used to differentiate among the subtypes, more markers have been shown to be associated with basal and luminal UCB in the above cited studies. Thus, additional tests with more immunohistochemical markers might offer different results. Results might also vary if a different scoring system instead of the IRS is applied for the quantification of marker expression. Moreover, in our cohort only 16 of the 222 patients (7.2%) were in the exclusively CK20 positive (CK20+/CK5-) subgroup, which appears to be a rare but a highly unfavorable subtype of UTUC. Analyses of larger cohorts of UTUC might offer different or more favorable results. Finally, since the cut-off points were explicitly defined in order to distinguish between patient subgroups with a good and worse prognosis, our results require independent validation.

## Conclusions

In conclusion, the present study demonstrates that UTUC can be divided into typical basal-like and luminal-like subtypes by use of immunohistochemistry even though a substantial number of the assessed cases did not follow this clear distinction. We were able to define four subtypes of UTUC by using CK5 and CK20 as surrogate markers for basal-like and luminal-like tumors. Finally, patients with exclusively CK20 positive expression (CK20+/CK5-) exhibited significantly worse cancer-specific survival compared to the other three subgroups. Further genetic research with larger cohorts and additional markers is necessary to grant further insight into the molecular subtyping of UTUC.

## Supporting information

S1 Fig Cancer-specific survival depending on subtype of UTUC defined by heatmap and cluster dendrogram on the expression of all seven markers. (TIF)

S2 Fig Cancer-specific survival depending on subtype of UTUC defined by heatmap and cluster dendrogram on CK5 and CK20 expression. (TIF)

S1 Table Minimal data set of the patient cohort with IRS scores for each of the seven markers. (XLSX)

S2 Table Internal validation of IRS cut-off values using LOO cross-validation and bootstrapping. (XLSX)
