# Supplementary figures and images for: Immunohistochemiocal subtyping using CK20 and CK5 can identify urothelial carcinomas of the upper urinary tract with a poor prognosis
Source: PLoS One. 2017 Jun 20;12(6):e0179602. doi: 10.1371/journal.pone.0179602 (PMC5478149; doi:10.1371/journal.pone.0179602)

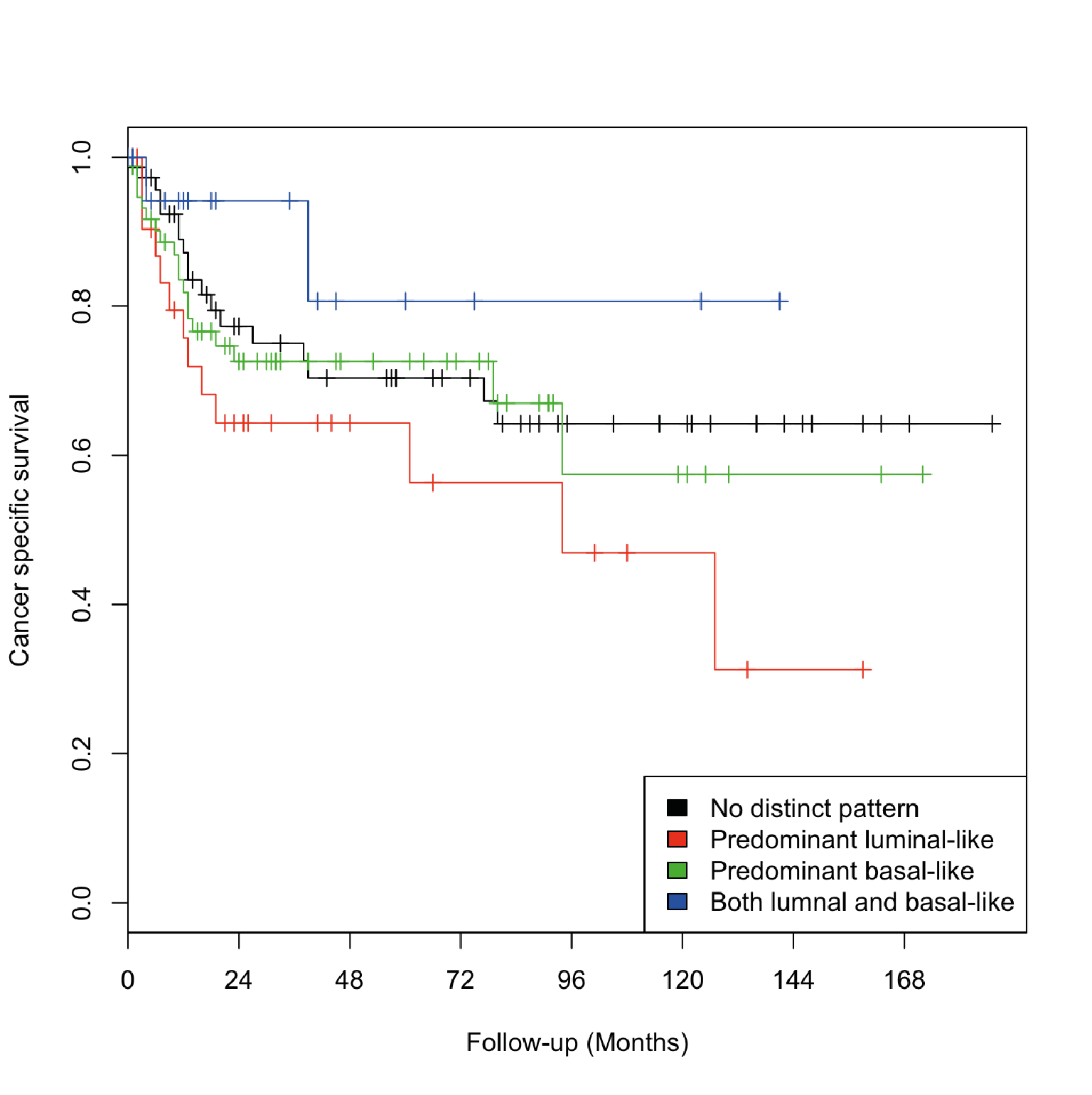

Supplement: S1 Fig — (TIF) [file pone.0179602.s001.tif]

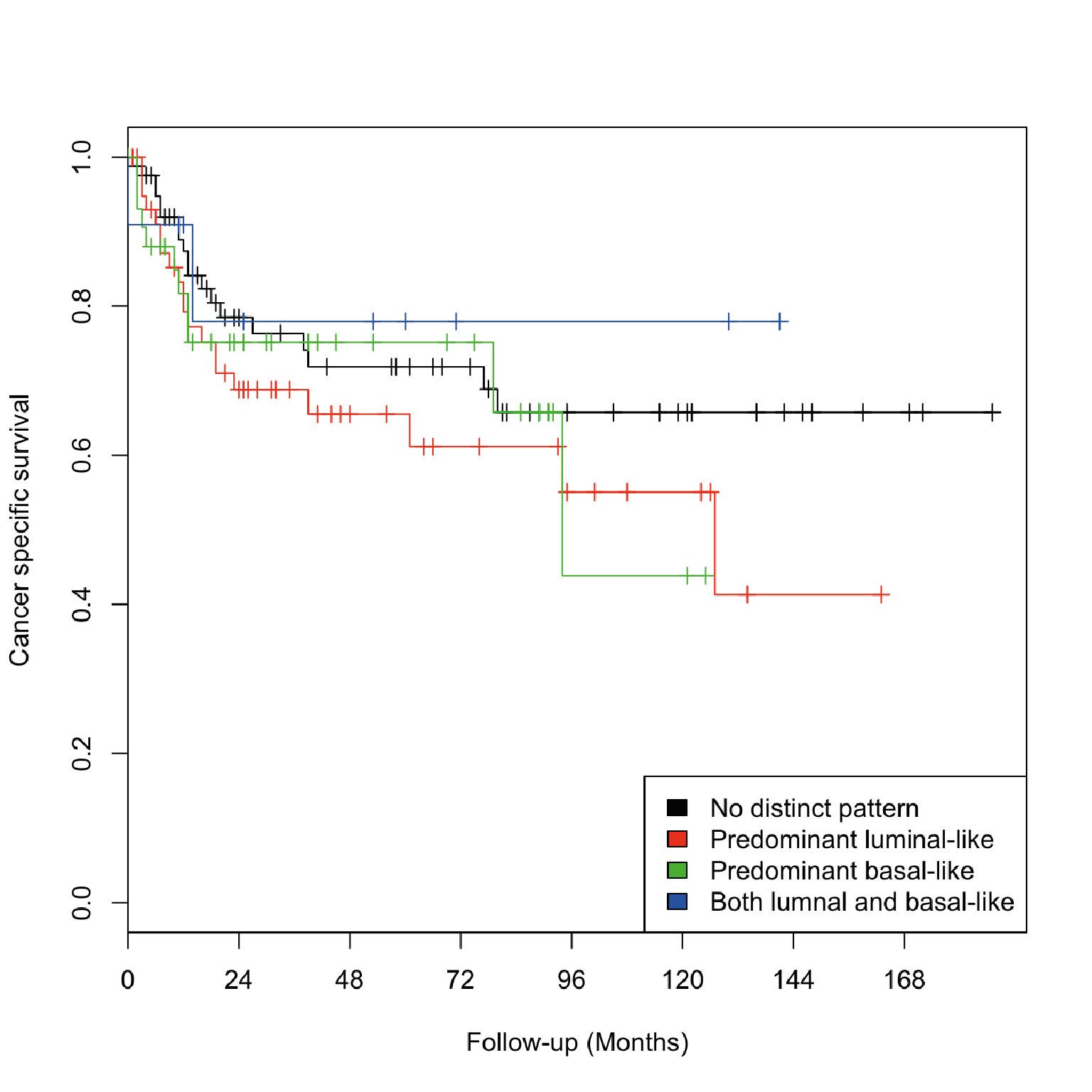

Supplement: S2 Fig — (TIF) [file pone.0179602.s002.tif]
